# Association between chronic obstructive pulmonary disease and biomass smoke in rural areas

**DOI:** 10.5588/ijtld.22.0337

**Published:** 2022-12-01

**Authors:** A. Ramírez-Venegas, F. Montiel-Lopez, J. L. Pérez Lara-Albisua, A. Aranda-Chávez, H. Perea-Gutiérrez, R. Falfán-Valencia, G. Pérez-Rubio, R. Pérez-Padilla, M. Ramírez-Díaz, M. L. Martínez-Gómez, F. Cruz-Vicente, I. Thirión-Romero, R. H. Sansores

**Affiliations:** 1 Department of Tobacco Smoking and COPD Research, Instituto Nacional de Enfermedades Respiratorias (INER) Ismael Cosío Villegas, Mexico City, Mexico; 2 Deparment of Traumatology and Othopedics, Hospital Central Norte Petróleos Mexicanos, Mexico City, Mexico; 3 Hospital General de Zona No.18, Insituto Mexicano del Seguro Social, Coahuila, Mexico; 4 HLA Laboratory, INER Ismael Cosío Villegas, Mexico City, Mexico; 5 Department of Epidemiological Survaillance, Jurisdicción Sanitaria 06 Sierra, Oaxaca, Mexico; 6 Anesthesiology Department, Hospital Regional de Alta Especialidad de Oaxaca, Oaxaca, Mexico; 7 Hospital General Dr Aurelio Valdivieso, Secretaria de Salud de Oaxaca, Oaxaca, Mexico; 8 Department of Respiratory Medicine, Medica Sur Clinic & Foundation, Mexico City, Mexico

Dear Editor,

In low- and medium-income countries (LMICs), chronic obstructive pulmonary disease (COPD) creates a high burden of morbidity and mortality in non-smokers.^1^ Because there is significant exposure to biomass burning in rural areas, the COPD burden could be more significant than in urban areas.^2^ Unfortunately, there are no health policies specific to rural areas designed to prevent, diagnose or treat COPD. The report presented here is part of a crossover study, with data from respiratory health campaigns carried out annually by the Tobacco and COPD Research Department of Instituto Nacional de Enfermedades Respiratorias (INER), Mexico.

The campaigns were held in rural areas of Mexico from 2013 to 2019 in seven villages of the Valle Region of Oaxaca (Santa Catarina Ixtepeji, Nuevo Zoquiapam, San Miguel del Río, San Pablo Macuiltianguis, San Bartolome Quialana, San Juan Guelavia, San Pablo Guila). The campaigns targeted women over 40 years old from the Zapotec indigenous group, who are exposed to biomass burning while cooking using firewood. The main objective of the campaigns was to detect COPD in these women, and to make them aware of the dangers associated with indoor air pollution. The campaign includes health education talks and educational materials (comics), in which the symptoms and damage caused by cooking with firewood are graphically represented. The comics were made under the supervision of medical researchers of the Tobacco and COPD Research Department. The campaigns in Oaxaca benefitted from being accompanied by health personnel who spoke their native dialect, which allowed clear messaging in the communities. COPD tests such as spirometry were performed by social service doctors (certified by the National Institute for Occupational Safety and Health Spirometry Training Programme). Pre and post bronchodilator spirometry was performed on each patient and only those with acceptable quality criteria were reported. Pulmonologists interpreted the spirometry data. Sociodemographic data on the following variables were collected: comorbidities, history of exposure to biomass using a respiratory questionnaire in which the cumulative exposure to biomass smoke was expressed in hour-years (the product of number of years of exposure, and average hours per day of exposure). When a person had a spirometry result consistent with COPD, she was given a brief educational talk about her condition, treated with a bronchodilator and referred to a health institution. Ethical approval for our study was not required, because the results were part of a retrospective investigation. Stata v14.0 (Stata Corporation, College Station, TX, USA) software was used for statistical analysis.

From 2013 to 2019, 680 women attended the campaigns, 581 of whom underwent spirometry; 498 (86%) women who met the spirometric quality criteria were analysed. The mean age was 60 ± 11 years (*P*=0.011), with mean height of 1.44 ± 0.07 m, mean weight of 61 ± 12 kg and mean body mass index (BMI) of 29 ± 5 kg/m^2^. There was no difference between women with COPD (WCOPD) and women without COPD (NONCOPD). The mean biomass exposure index was 228 ± 157 hours/year. Using forced expiratory volume in 1 sec/forced vital capacity (FEV_1_/FVC) <0.70 as cut-off, COPD prevalence was 9.6%; based on the lower limit of normality (LLN), this increased to 11.6%. Of the WCOPD, 50% were classified as GOLD (Global Initiative for Chronic Obstructive Lung Disease) I, and 83% as group B.^3^ There was no difference in comorbidities such as asthma (4%), tobacco smoking (1%), high blood pressure (24%) or obesity (40%) between WCOPD and NONCOPD participants, except for diabetes mellitus, which showed higher prevalence in NONCOPD women (*P* = 0.022). In general, WCOPD had more respiratory symptoms, higher mMRC (modified Medical Research Council) score and CAT (COPD Assessment Test) score than NONCOPD women (Table). WCOPD presented more wheezing than NONCOPD (*P* < 0.001). Using logistic regression analysis and after adjusting for age and BMI, only the history of dyspnoea (OR 2.4, 95% CI 1.13–5.10; *P*=0.022) was found to be a risk factor for COPD.

**Table:** Pulmonary function, respiratory symptoms, quality of life, and physical examination data on women with COPD (by fixed ratio) and women without COPD, Oaxaca, Mexico

Variable	WCOPD (*n* = 48, 9.6%) *n* (%)	NONCOPD (*n* = 450, 90.4%) *n* (%)	Total (*n* = 498) *n* (%)	*P* value
Pulmonary function, mean ± SD				
FVC% pred	87 ± 28	96 ± 18	95 ± 19	0.002
FEV_1_% pred	74 ± 25	102 ± 18	99 ± 21	<0.001
FEV_1_/FVC	0.61 ± 0.11	0.83 ± 0.06	0.81 ± 0.09	<0.001
SpO_2_	94 ± 3	95 ± 3	95 ± 3	0.073
Symptoms, quality of life and physical examination				
Respiratory symptoms	24 (63)	132 (49)	156 (51)	0.100
Cough >3 months in the last year	10 (26)	22 (8)	32 (10)	0.001
History of dyspnoea in the last 3 months	21 (55)	81 (30)	102 (33)	0.002
History of wheezing in the last 3 months	14 (37)	52 (19)	66 (21)	0.013
mMRC score, mean ± SD	0.60 ± 0.76	0.49 ± 0.85	0.50 ± 0.87	0.207
CAT score, mean ± SD	6 ± 5	3 ± 4	4 ± 4	0.005
Wheezing	6 (16)	7 (3)	13 (4)	<0.001
Crackling	3 (8)	6 (2)	9 (3)	0.052

WCOPD = women with chronic obstructive pulmonary disease; NONCOPD = women without chronic obstructive pulmonary disease; SD = standard deviation; FVC% pred = forced volume capacity percentage of predicted; FEV_1_%pred = forced expiratory volume in the first second percentage of predicted; SpO_2_ = oxygen saturation; mMRC = modified Medical Research Council; CAT = COPD Assessment Test; COPD = chronic obstructive pulmonary disease.

These respiratory health campaigns in rural and marginalised areas indicate a high prevalence of COPD among women (either based on FEV_1_/FVC fixed ratio, or on the LLN). In comparison to PLATINO,^4^ the prevalence of COPD in these rural areas was almost twice that reported in Mexico City, where the prevalence was 7.8% with FEV_1_/FVC fixed ratio and 5.7% with LLN, and higher than the PREPOCOL study (8.4%).^5^ The high prevalence of COPD in the Valle of Oaxaca also contrasts with the low prevalence of COPD associated with biomass burning in a suburban area of Mexico City.^4^ This suggests that the use of biomass, alongside increased levels of poverty, results in more cases in rural areas. Prevalence estimates based on LLN are generally lower than the fixed ratio. In this study, the higher COPD prevalence based on LLN may be explained by the fact that relatively younger patients (age <50 years) were included. Recent data suggest that the high prevalence of COPD in many LMICs reflects the high prevalence of risk factors such as exposure to biomass smoke.^1^,^4^–^6^ Even after adjusting for income variations, the prevalence of COPD in LMICs based on LLN was marginally higher in women (7.4%) than in men (7.1%).^4^ This is a gender-associated disease, because women spend longer by the stove in poorly ventilated spaces.

Both in our study and in the PLATINO research,^4^,^7^ a high proportion of symptoms was observed; however, WCOPD in this study presented more symptoms (dyspnoea, wheezing, cough) than in the PLATINO study.^7^ For example, in our study 47% of women reported dyspnoea, whereas only 30% reported this in the PLATINO study. Unfortunately, women did not associate their symptoms, particularly dyspnoea, with exposure to biomass smoke in either of these studies. In the absence of spirometers in rural areas, our report shows how the respiratory symptoms questionnaires helped to identify people who might have COPD associated with biomass use by physicians in rural clinics.^8^

There have been some isolated actions regarding educational campaigns focused on COPD and tobacco smoking in rural areas.^6^,^9^ However, exposure to biomass smoke was not considered within the scope of the campaign. Our results show that health campaigns can diagnose COPD in a high proportion of women in rural areas and make them aware of the respiratory symptoms and risks associated with chronic exposure to wood smoke. In these campaigns, almost 700 women received health educational materials and educational talks, and underwent a respiratory health and spirometry check-up. COPD resulting from biomass smoke is among the most neglected diseases globally, receiving little attention from healthcare providers. Although some initiatives to address COPD have emerged,^10^ greater effort is needed to focus on LMICs where the burden of COPD associated with biomass use remains high.

## References

[1] Adeloye D (2022). Global, regional, and national prevalence of, and risk factors for, chronic obstructive pulmonary disease (COPD) in 2019: a systematic review and modelling analysis. Lancet Respir Med.

[2] Ramírez-Venegas A (2018). Prevalence of COPD and respiratory symptoms associated with biomass smoke exposure in a suburban area. COPD.

[3] Global Initiative for Chronic Obstructive Lung Disease (2021). 2021 GOLD Reports. GOLD. https://goldcopd.org/2021-gold-reports/.

[4] Menezes AMB (2005). Chronic obstructive pulmonary disease in five Latin American cities (the PLATINO study): a prevalence study. Lancet.

[5] Caballero A (2008). Prevalence of COPD in five Colombian cities situated at low, medium, and high altitude (PREPOCOL Study). Chest.

[6] Yuan X (2015). Long-term efficacy of a rural community-based integrated intervention for prevention and management of chronic obstructive pulmonary disease: a cluster randomized controlled trial in China’s rural areas. Braz J Med Biol Res.

[7] Nonato NL (2015). Occurrence of respiratory symptoms in persons with restrictive ventilatory impairment compared with persons with chronic obstructive pulmonary disease: the PLATINO study. Chron Respir Dis.

[8] Bergna MA (2015). Development of a simple binary response questionnaire to identify airflow obstruction in a smoking population in Argentina. Eur Respir Rev.

[9] Nagourney EM (2020). Illness representations of chronic obstructive pulmonary disease (COPD) to inform health education strategies and research design-learning from rural Uganda. Health Educ Res.

[10] Halpin DMG (2019). The GOLD Summit on chronic obstructive pulmonary disease in low- and middle-income countries. Int J Tuberc Lung Dis.

